# High-speed imaging of ESCRT recruitment and dynamics during HIV virus like particle budding

**DOI:** 10.1371/journal.pone.0237268

**Published:** 2020-09-04

**Authors:** Shilpa Gupta, Josh Bromley, Saveez Saffarian

**Affiliations:** 1 Center for Cell and Genome Science, University of Utah, Salt Lake City, Utah, United States of America; 2 Department of Biology, University of Utah, Salt Lake City, Utah, United States of America; 3 Department of Physics and Astronomy, University of Utah, Salt Lake City, Utah, United States of America; University of Alabama at Birmingham, UNITED STATES

## Abstract

Endosomal sorting complexes required for transport proteins (ESCRT) catalyze the fission of cellular membranes during budding of membrane away from the cytosol. Here we have used Total Internal Reflection Fluorescence (TIRF) microscopy to visualize the recruitment of ESCRTs specifically, ALIX, CHMP4b and VPS4 onto the budding HIV Gag virus-like particles (VLPs). We imaged the budding VLPs with 200 millisecond time resolution for 300 frames. Our data shows three phases for ESCRT dynamics: 1) recruitment in which subunits of ALIX, CHMP4b and VPS4 are recruited with constant proportions on the budding sites of HIV Gag virus like particles for nearly 10 seconds, followed by 2) disassembly of ALIX and CHMP4b while VPS4 signal remains constant for nearly 20 seconds followed by 3) disassembly of VPS4. We hypothesized that the disassembly observed in step 2 was catalyzed by VPS4 and powered by ATP hydrolysis. To test this hypothesis, we performed ATP depletion using (-) glucose medium, deoxyglucose and oligomycin. Imaging ATP depleted cells, we show that the disassembly of CHMP4b and ALIX observed in step 2 is ATP dependent. ATP depletion resulted in the recruitment of approximately 2-fold as many subunits of all ESCRTs. Resuming ATP production in cells, resulted in disassembly of the full ESCRT machinery which had been locked in place during ATP depletion. With some caveats, our experiments provide insight into the formation of the ESCRT machinery at the budding site of HIV during budding.

## Introduction

The Endosomal Sorting Complexes Required for Transport (ESCRT) catalyze the fission of membrane during budding away from the cytosol [1]. As such, ESCRTs play an essential role in multivesicular body formation [2], HIV budding [3–5], exosome release [6] and cytokinesis [7, 8], among others. The assembly of ESCRTs on the cytosolic front of the membrane buds is perplexing, since the ESCRT assembly which localizes within the shrinking bud neck, would presumably get in its own way during the catalysis of the membrane. It is also plausible that ESCRTs assemble away from the actual membrane scission location and scission is catalyzed by phase transitions driven by lipids enriched at the budding site. Regardless of the mechanism of fission, how ESCRTs organize during the scission remains to be understood [9–12].

During HIV budding, the p6 domain of Gag [13, 14] which encodes two late domain motifs, interacts with ESCRT elements TSG101 [15–17] and ALIX [18–20]. ALIX was shown to also interact with the Gag NC domain [21, 22] as well as CHMP4b which is a member of ESCRT-III proteins [23]. It was also shown that overexpression of ESCRT-III proteins results in assembly of a spiral form on the plasma membrane which protrudes the membrane outward from the cytosol [24]. Also, purified ESCRT-III proteins have a tendency of decorating lipid tubes as concentric helices [25]. CHMP4 proteins also interact with VPS4 which is the only AAA ATPase within the ESCRT family [26]. It has been shown in vitro that the addition of VPS4 and ATP can drive de-polymerization of ESCRT-III proteins decorating membrane tubules [27–29]. While a lot of structural data exists on individual components of the ESCRT pathway, how these proteins come together to catalyze the membrane fission is still not well understood.

We have previously shown that any delay in the recruitment of ESCRTs during HIV budding results in the release of non-infectious HIV virions due to activation of the HIV protease before virion release and loss of important HIV enzymes [30]. The HIV Gag expression within the cytosol, without any other viral factors, results in assembly and budding of virus like particles with the same size as full-length HIV [31]. Recruitment of ESCRT proteins during budding of HIV Gag VLPs has been studied extensively using live imaging by our group and others [9, 32–35]. Similar results are obtained when ESCRT recruitment is imaged during HIV particle assembly, where particles incorporate both Gag and Gag-Pol [36–39]. The consensus emerging from these experiments is that ALIX, CHMP4b and VPS4 are recruited at the end of HIV Gag assembly for a short period of one or two minutes. The total duration of the recruitment of ESCRTs which takes ~1–2 minutes, is far shorter than the time it takes to assemble an HIV virion or virus-like particle both of which take between 10–20 minutes from nucleation to ESCRT recruitment [33, 39, 40].

Due to the photo-toxicity and impacts on cell health during imaging, it has been challenging to image the HIV assembly and ESCRT recruitment with high-speed imaging. High-speed imaging data is however essential to dissect the relative recruitment of the ESCRT components. In this manuscript, we have used three color imaging to identify assembling HIV Gag VLPs on the plasma membrane and image the recruitment of ESCRT elements ALIX, CHMP4b and VPS4 with 200 millisecond (ms) temporal resolution. Our experiments provide insight into the formation of the ESCRT machinery at the site of HIV Gag during budding.

## Materials and methods

### Cell culture and transfection

HeLa cells and HEK 293 were maintained in Dulbecco’s modified Eagle’s medium (DMEM; Invitrogen, Carlsbad, CA) supplemented with fetal calf serum (10%), sodium pyruvate (1 mM) and L-glutamine (2 mM). HeLa cell lines stably expressing eGFP-tagged ALIX were maintained in the same medium supplemented with G-418 (0.5 mg/mL) for selection. For TIRF experiments, cells were incubated in CO2-independent medium (LifeTechnologies) in the case of WT condition.

Cells were seeded 18 hours before transfection on sterile 4 chamber dishes at 60% confluence. Transfection was carried out using Lipofectamine2000 (Invitrogen) and DNA plasmid at the ratio of 3:1 with total DNA of 1500 ng. For ALIX-VPS4 study, cells were transfected with 1200 ng of VPS4 plasmid and 300 ng of HIV Gag plasmid. For CHMP-VPS4 study, cells were transfected with 450 ng of CHMP plasmid, 750 ng of VPS4 plasmid and 300 ng of HIV Gag plasmid. For ALIX-CHMP study, cells were transfected with 900 ng of CHMP plasmid and 600 ng of HIV Gag plasmid. Cells were used for imaging 6–7 hours after transfection. The cells were kept at 37°C during the imaging.

### Microscopy

Live images were acquired using iMIC Digital Microscope made by TILL photonics controlled by TILL’s Live Acquisition imaging software as previously described [27] using Andor iXon camera. Two wavelengths of laser, 488 nm diode laser (Coherent, Saphire 488) and 561 nm diode-pumped solid state (DPSS) laser (Cobolt Jive, 561 nm Jive High Power), were used to excite eGFP and mCherry, respectively. 60X objective was used for the experiments. Laser beams passed through an AOTF (acousto-optical tunable filter) and focused into a fiber which delivers the light to TILL Yanus digital scan head and then Polytrope II optical mode switch. Polytrope hosts a quadrant photodiode used for TIRF penetration depth calibration, which was set to 150 nm for the experiments in this manuscript. Once the penetration depths for the experiments are set at the beginning of acquisition, a feedback loop keeps the focus of the objective on the sample by constantly monitoring the position of the back reflected beam with respect to the original beam.

### Microscopy data analysis

Images from the microscope were stored as TIFF files and analyzed using Matlab software (Mathworks) as described previously [32] and deposited in (https://github.com/saveez/SaffarianLab). Intensity of the fluorescent signal collected from each diffraction limited spot is proportional to the number of molecules within that position, however the intensity is also proportional to the laser intensity, position of molecules with respect to glass during TIRF and substitution level of WT versus fluorescent molecules in each particular cell. To compare intensities of the ESCRT recruitments in between various cells and experimental conditions, the average of the actual intensities were normalized to 10000 a.u. The rate of assembly and disassembly are calculated using Boltzman Growth Equation after fitting the recruitment dynamics.

### ATP depletion-repletion

After imaging of cells with WT condition is completed, the medium was discarded and cells were washed three times using PBS. Cells were then incubated in Dulbecco's modified Eagle's medium with no glucose supplemented with 40 mM deoxyglucose and 2.5 mM oligomycin. After imaging for 10 minutes, this medium was discarded and normal DMEM medium put back after thrice washing cell with PBS to supply cells with ATP.

### Western blot analysis

Virion and cell lysates were separated on 4–15% polyacrylamide gels and transferred to Immobilon-FL membranes. The blots were probed with anti-p24 (183-H12-5C, NIH AIDS Reagent Program), anti-eGFP (Santa Cruz), and infrared dye coupled secondary antibodies (LI-COR) were used for immunoprobing. Scanning was performed with the Odyssey infrared imaging system (LI-COR) in accordance with the manufacturer’s instructions at 700 or 800 nm, accordingly.

### Infectivity assay

HEK 293T cells (60% confluent in 4 cm plates) were transfected using lipofectamine-2000 with NL4.3 vector alone or along with ΔCMV-eGFP-flex-CHMP4b plasmid. The supernatant was harvested 48 hours later. Infectivity was measured by adding the supernatant to TZM-B1 cells (80% confluent). 48 hours later cells were lysed using britelite plus Reporter Gene Assay (Perkin Elmer) and luminosity was measured using a Cytation 5 microscope, experiments were carried out in triplicate.

### Statistics

All conditions tested in the manuscript contain nearly 40 virus like particles analyzed from 20–30 cells except for ATP depletion-repletion study where 10 virus like particles analyzed from 2 cells. The experiments were performed at least 2 times. There was no data selection applied to the sample, therefore all relevant data collected from the microscopy were analyzed and plotted in the figures.

### Cell lines

The HeLa and HEK 293T cells were obtained from ATCC. TZM-b1 cells were acquired from NIH AIDS Reagent Program.

## Results

### Recruitment dynamics of ALIX and VPS4 into budding HIV Gag VLPs

**Fig 1:**
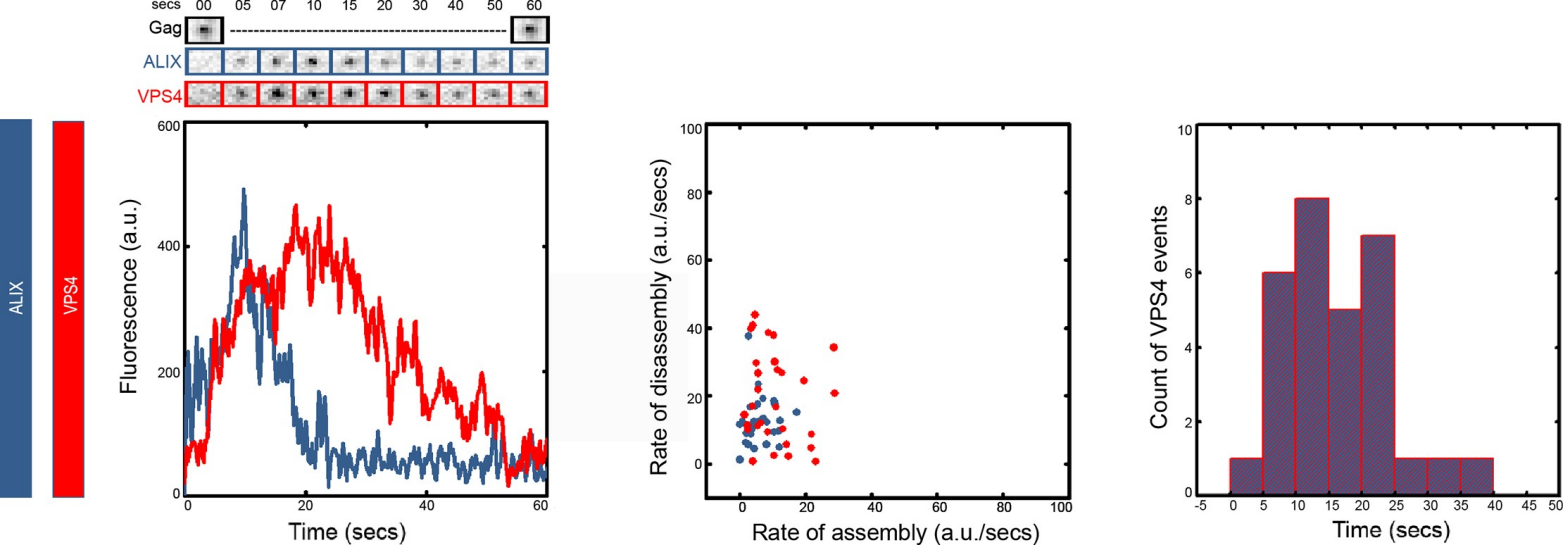
ALIX is disassembled in the presence of VPS4. The left graph shows the intensity plot of ALIX (Blue) and VPS4 (Red) and cropped TIRF images of the Gag (topmost, Black), ALIX (middle, Blue) and VPS4 (bottom, Red). The scatter plot in the middle panel shows the rate of assembly and disassembly for ALIX and VPS4. The histogram on the right panel shows the distribution of the retention time of VPS4. (Rates of assembly/disassembly as well as the retention time are defined in S2 Fig).

To image the recruitment dynamics of ESCRTs, relative to each other, onto budding HIV Gag VLPs we imaged recruitment of two fluorescently tagged ESCRT proteins into fluorescently tagged HIV Gag VLPs. We used three color TIRF imaging to visualize the proteins of interest. HeLa cells stably expressing ALIX-h30-eGFP were used which are termed as HeLa-C1 cells and were previously characterized in our lab [32]. ALIX-h30-eGFP links ALIX with eGFP through a stiff 30 amino acid super helical linker and is functional with the same efficiency as WT in rescue of PTAP^-^ HIV virions. These cells were transfected with a mixture of HIV Gag-BFP [41] and VPS4-h37-mcherry plasmids (previously characterized in [33]) as explained in methods. Cells were imaged after the membrane was full of formed VLPs approximately 6-10hrs after transfection. The imaging was conducted for 1 minute with a frame rate of 200 ms / frame in both 488 and 561 channels corresponding to ALIX-h30-eGFP and VPS4-h37-mcherry. To reduce photo-toxicity, we took advantage of the limited movement of the Gag VLP during the one minute of imaging and only imaged the Gag-BFP at the start and at the end of the total imaging period. We analyzed the recruitment of ALIX and VPS4 in nearly 40 VLPs from 30 cells. We imaged each cell only once. The 1 minute imaging window does not allow observation of recruitment events in all observed VLPs, however a subset of VLPs in each cell were found that recruited ESCRTS. As shown in Fig 1, ALIX and VPS4 get recruited simultaneously with VPS4 having a small delay in reaching its final accumulation in all the 40 VLPs analyzed. In 80% of the 40 VLPs analyzed, ALIX disassembles while VPS4 platform remains for nearly 10 more seconds before its disassembly. The rest 20% shows ALIX disassembly together with VPS4 (S1 Fig). We focused on the major phenotype and took those data for further calculations. We analyzed the rates of assembly and disassembly based on a sigmoidal model as presented in S2 Fig, we found that VPS4 assembles almost twice slower than ALIX (rate of assembly for VPS4 is 11 ± 8 a.u./sec, whereas, for ALIX it is 6 ± 4 a.u./sec) and disassembles with similar rate to ALIX (rate of disassembly for VPS4 is 19 ± 13 a.u./sec and for ALIX it is 13 ± 7 a.u./sec). On calculating the time of retention of VPS4, we found that VPS4 stays for 15 ± 8 seconds after full recruitment, which is significantly more than ALIX as shown in Fig 1.

### Recruitment dynamics of CHMP4b and VPS4 into budding HIV Gag VLPs

To image the recruitment dynamics of CHMP4b with respect to VPS4 onto budding HIV Gag VLPs, Hela cells were transiently transfected with a mixture of HIV Gag-BFP, VPS4-h37-mcherry and eGFP-flex-CHMP4b plasmids as explained in methods. We created a plasmid which expresses human CHMP4b linked to eGFP by a flexible linker at its N-terminus under a ΔCMV promoter (ΔCMV-eGFP-flex-CHMP4b). A similar N-terminally tagged CHMP4b has been used before to visualize recruitment of CHMP4b onto the assembling Gag VLPs by other labs [35]. The co-expression of this plasmid had no effect on the release of HIV Gag VLPs as shown in S3 Fig. In addition as shown in S3 Fig, we further characterized this plasmid in infectious HIV release and found a slight decrease in virion release with no effect on infectivity of the released virions.

**Fig 2:**
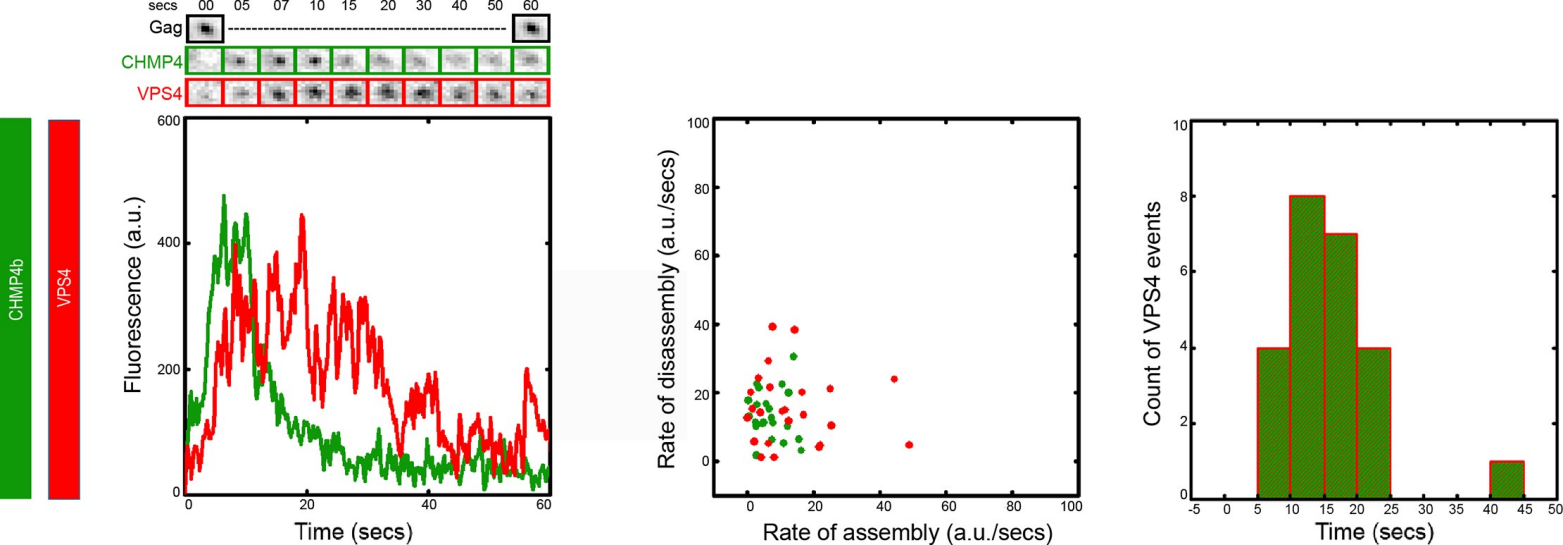
CHMP4b is disassembled in the presence of VPS4. The left panel shows the intensity plot of CHMP4b (Green) and VPS4 (Red) and cropped TIRF images of the Gag (topmost, Black), CHMP4b (middle, Green) and VPS4 (bottom, Red). The scatter plot in the middle panel shows the rate of assembly and disassembly for CHMP4b and VPS4. The histogram on the right panel shows the distribution of the timing of retention of VPS4. (Rates of assembly/disassembly as well as the retention time are defined in S2 Fig).

Transfected HeLa cells were imaged after the membrane was full of formed VLPs. The imaging was conducted similarly as the above section. We analyzed the recruitment of CHMP4b and VPS4 in 40 VLPs from 25 cells. As shown in Fig 2, CHMP4b and VPS4 get recruited simultaneously with a slight delay of VPS4 in attainment of its final accumulation in all the 40 VLPs analyzed. In 70% of the 40 VLPs analyzed, CHMP4b disassembles while VPS4 platform remains for nearly 10 more seconds before its disassembly (similar phenotype to ALIX and VPS4). The rest 30% of events show CHMP4b and VPS4 disassembly together (S1 Fig). We focused on the major phenotype and took those data for further calculations. On analyzing the rate of assembly and disassembly, we found that VPS4 assembles almost twice slower than CHMP4b (rate of assembly for VPS4 is 13 ± 12 a.u./sec, whereas, for CHMP4b it is 7 ± 5 a.u./sec) and disassembles with nearly similar rate to CHMP4b (rate of disassembly for VPS4 is 16 ± 10 a.u./sec and for CHMP4b it is 18 ± 22 a.u./sec). On calculating the time of retention of VPS4, we found that VPS4 stays for 15 ± 7 secs after getting recruited, which is more than 10 seconds longer than the CHMP4b. This data shows similar de-polymerization of ALIX and CHMP4b in the presence of VPS4.

### Recruitment dynamics of ALIX and CHMP4b into budding HIV Gag VLPs

**Fig 3:**
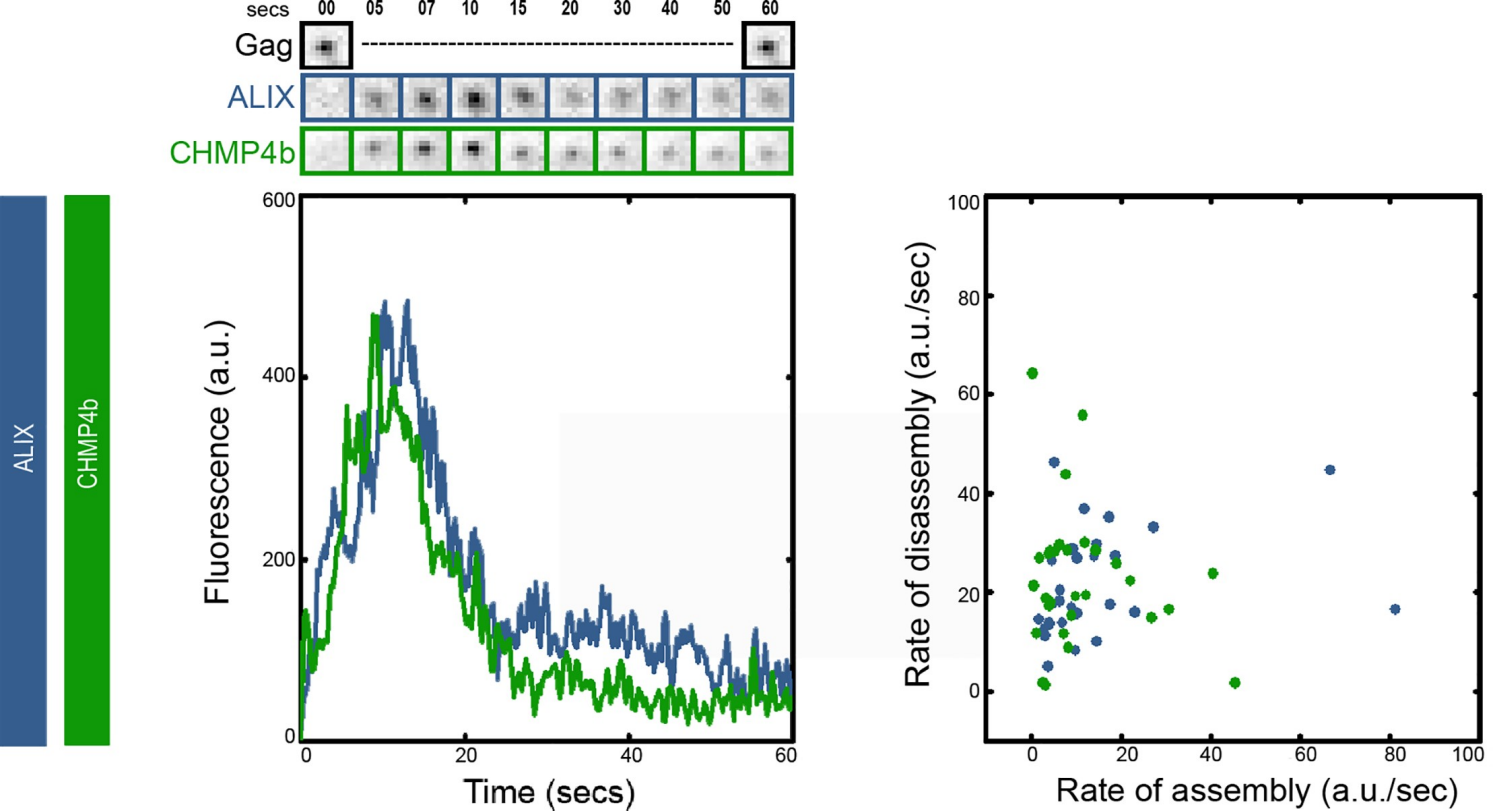
ALIX and CHMP4b assemble and disassemble together. The left graph shows the intensity plot of ALIX (Blue) and CHMP4b (Green) and cropped TIRF images of the Gag (topmost, Black), ALIX (middle, Blue) and CHMP4b (bottom, Green). The scatter plot on the right shows the rate of assembly and disassembly for ALIX and CHMP4b. (Rates of assembly/disassembly are defined in S2 Fig).

The data presented in the previous sections shows that VPS4 plateaus while ALIX or CHMP4b is disassembled. To visualize the recruitment dynamics of CHMP4b with respect to ALIX onto budding HIV Gag VLPs, we used HeLa-R1 cells (constitutively expressing ALIX-h30-mcherry) and transiently transfected them with a mixture of HIV Gag-BFP, and eGFP-flex-CHMP4b plasmids as explained in methods. The imaging was conducted similarly as above sections. We analyzed the recruitment of CHMP4b and ALIX in 40 VLPs from 20 cells. As shown in Fig 3, CHMP4b and ALIX get assembled and disassembled together in all the 40 VLPs analyzed. On analyzing the rate of assembly and disassembly, we found that ALIX and CHMP4b have similar rates for both assembly (rate of assembly for ALIX is 14 ± 17 a.u./sec, whereas, for CHMP4b it is 12 ± 15 a.u./sec) and disassembly (rate of disassembly for ALIX is 20 ± 10 a.u./sec and for CHMP4b it is 22 ± 14 a.u./sec). The total assembly time was also similar between CHMP4b and ALIX (10 ± 4 seconds). Our result indicate that ALIX and CHMP4b are assembled and disassembled together.

### Recruitment dynamics of CHMP4b and VPS4 into budding HIV Gag VLPs during ATP depletion and repletion

**Fig 4:**
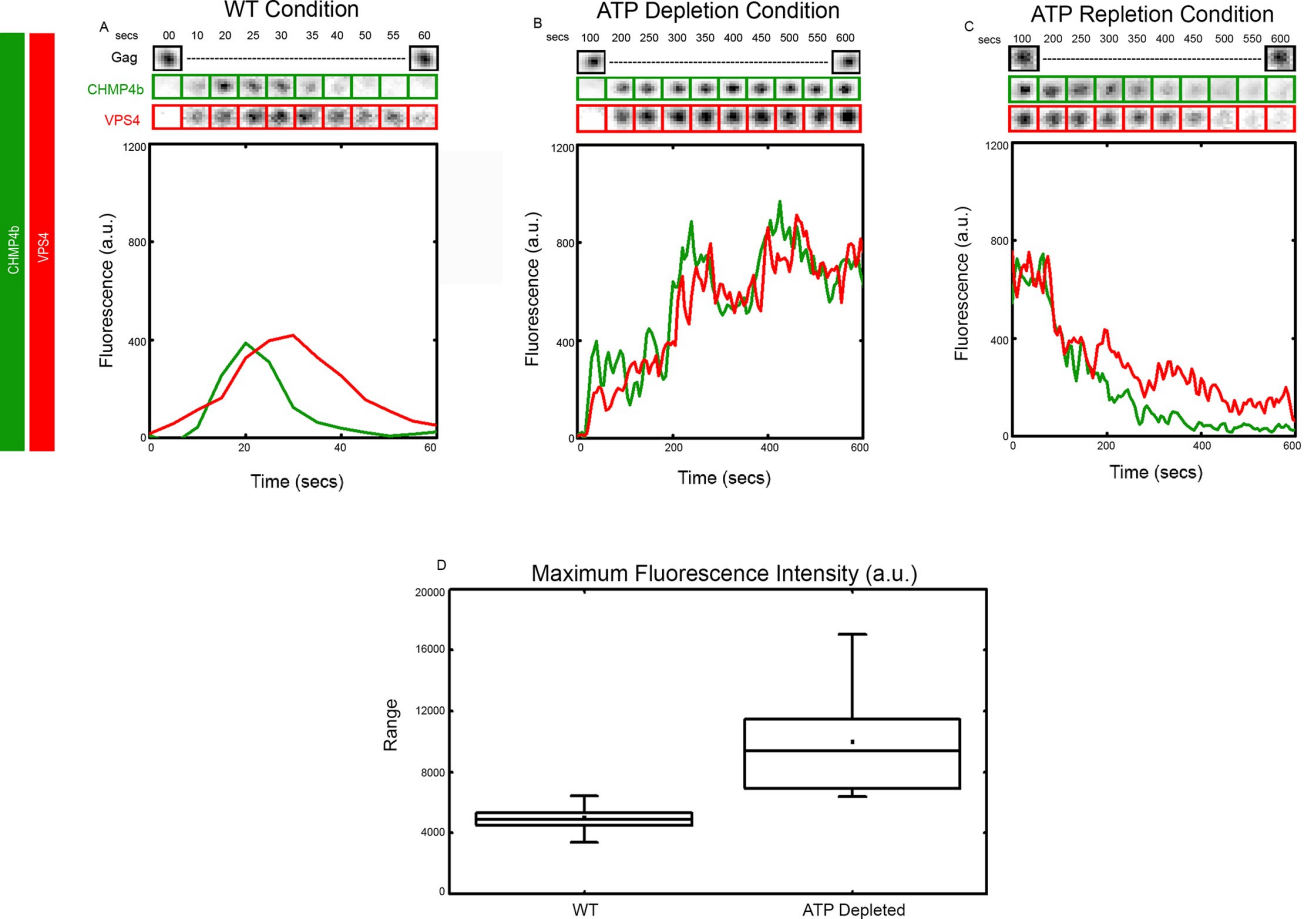
Dynamics of CHMP4b and VPS4 during ATP depletion and repletion. Recruitment of CHMP4b (Green) and VPS4 (Red) shown by plotting intensity and showing cropped TIRF images of the Gag (topmost, Black), CHMP4b (middle, Green) and VPS4 (bottom, Red) in (A) WT condition, (B) ATP depleted condition and (C) ATP repletion condition. Box plot compares the normalized maximum intensity of ESCRTS in WT and ATP-depleted condition (D).

Next, we imaged cells during ATP depletion and visualized the recruitment of CHMP4b and VPS4 into HIV Gag VLPs. Hela cells were transiently transfected with a mixture of HIV Gag-BFP, VPS4-h37-mcherry and eGFP-flex-CHMP4b plasmids. Cells were first imaged at a reduced frame rate of one frame/ 5 seconds to reduce exposure and toxicity in their WT condition to detect normal recruitment events for 1 minute at which point the medium was removed and replaced with the ATP depletion cocktail as described in the methods. The same cells were then imaged for 10 minutes at which point all recruited ESCRTs were locked on their corresponding VLPs. The ATP depletion medium was removed at this point and cells were returned to normal imaging medium and imaged for an additional 10 minutes. We analyzed the recruitment of CHMP4b and VPS4 in 10 VLPs from 2 cells for different conditions. As shown in Fig 4A, CHMP4b and VPS4 get recruited together with a slight delay of VPS4 as expected in WT scenario. As expected, CHMP4b gets disassembled before the disassembly of VPS4. During ATP depletion phase, both CHMP4b and VPS4 get recruited in a similar fashion onto the VLPs and get stuck (constant fluorescence intensity over time) as shown in Fig 4B. On analyzing the normalized maximum intensity of ESCRTs in WT and ATP-depleted condition, we found that almost twice the amount of ESCRTs are recruited when cells get depleted of ATP (4900 ± 1300 a.u. vs 9900 ± 3300 a.u.), shown in Fig 4D. On repletion of ATP the recruited ESCRT structures are disassembled again with CHMP4b depolymerizing slightly faster than VPS4 as shown in Fig 4C, indicating that the ESCRTs reverted to their normal mechanism upon ATP repletion.

## Discussion

We present ultrafast imaging of ALIX, CHMP4b and VPS4 on budding HIV virus like particles. Our data shows that ALIX, CHMP4b and VPS4 are recruited concurrently for 10 ± 4 seconds after initiation of ESCRT assembly which is considered to be phase I (S2 Fig). After the initial assembly, VPS4 proteins remain associated with the VLP, while CHMP4b and ALIX are disassembled in a linear fashion in the phase II (S2 Fig). After about 10 seconds, the assembled VPS4 also starts disassembling with a nearly similar rate to CHMP4b and ALIX. This phase is termed as phase III (S2 Fig). While our observation on ALIX are completely novel, the observed behavior of VPS4 and CHMP4b is consistent with live imaging experiments on HIV Gag VLPs by Beck et al. which were performed at 10 seconds/frame and showed a time gap between disappearance of CHMP4b and VPS4 signals [34]. We hypothesized that the VPS4 proteins during the phase II of the assembly had formed a functioning VPS4 platform that depolymerizes the CHMP4b and ALIX proteins using ATP hydrolysis. This hypothesis is supported by the experiments in ATP depleted cells, where assembly of VPS4 and CHMP4b occurs and continues until approximately double the usual amount of these proteins is accumulated on the VLP. Repletion of ATP results in a linear de-polymerization of the accumulated CHMP4b proteins, followed by a short delay of VPS4.

While we will be discussing the potential caveats of our imaging approach extensively in this discussion, it is worth noting that ATP depletion experiments are challenging and we present a lower number of VLPs analyzed under ATP depletion compared to normal conditions, however while the number of our events are low, we present data, before, during and post ATP depletion from the same VLPs which is essential. Our observations suggest a role for ATP hydrolysis during the de-polymerization of CHMP4b by VPS4. These observations are in general agreement with *in vitro* studies of ESCRT-III and VPS4 assemblies on lipid tubules [12, 27–29]. They are also consistent with imaging of assembled CHMP4 filaments in cells depleted of VPS4 [42], it is important to highlight that our ATP depletion experiments are transient and take effect within a few minutes, while VPS depletion takes a few days. Our observation differs significantly with imaging results from yeast MVBs. Ultrafast imaging in yeast showed a continuous assembly and disassembly of VPS4 and ESCRT-III proteins independent of the presence of ATP [43]. It is unlikely that VPS4 and ESCRT-III proteins behave differently in the MVB pathway and HIV budding and therefore, this difference would remain to be further studied. Also, in vitro studies in GUVs have highlighted the role of ATP in force generation required for membrane scission [44]. It is important to point out that the membrane curvature, size and number of molecules involved in the in vitro experiments, are much larger than the sub-diffraction limited budding necks observed in our study.

Technical difficulties in ultrafast imaging precluded us from measuring the moment of VLP release. It has been recently suggested that the virions are released 10’s of seconds after disassembly of ESCRTs [9]. In this study, we have acquired the ultrafast imaging capability to observe the dynamics of ESCRTs and specifically ATP dependent disassembly of CHMP4b and VPS4, however, we still do not have the technical capability to detect the release of the virions with the same temporal resolution. The capability of ultrafast imaging of ESCRTs in combination with the detection of VLP release and depletion of ATP would be an essential technical milestone to be developed in the future. It is of note that in principle one can detect the approximate moment of membrane scission by observing the change in the movement of VLPs post release as previously shown [38, 39], however, in our setup, the Gag molecules were labeled with BFP which precluded rapid imaging of VLPs required for detection of approximate moment of scission.

Our observation that ATP depletion results in a lock up of ESCRT machinery on the budding HIV virions provides an important tool for the ultra-structural characterization of the ESCRT machinery. We point out that our observations are performed on a limited number of cells and VLPs due to technical difficulties, however while the number of our events are low, we present data, before, during and post ATP depletion from the same VLPs which is essential. As mentioned before, the ultra-structural arrangement of the ESCRTs within the neck of the budding vesicles is unknown. It is plausible to use ATP depletion as a tool to lock up the ESCRTs prior to their ultra-structural analysis using either cryotomography [45] or high resolution optical imaging [34, 36, 46].

Our imaging conditions are imperfect and can have caveats: While we have performed functional assays to make sure that our fluorescently tagged ESCRTs behave similarly to the wild type (S3 Fig and [32, 33]) we are still expressing the ESCRT proteins in addition to the WT proteins which will result in a mild over expression of ESCRTs. How does this over expression affect the dynamic of ESCRT remains to be investigated. We are working towards creating complete genomic replacement of ESCRTs with their fluorescently tagged counterparts. Experiments in these new cell lines will both resolve issues related to over expression as well as allow faithful counting of ESCRT components during their assembly for catalyzing the fission of membrane.

## Supporting information

S1 Fig Minor phenotype of CHMP4B, ALIX and VPS4 recruitment. Left panel shows ALIX (Blue) and VPS4 (Red) signal during recruitment event onto HIV Gag-BFP VLP. Right panel shows CHMP4B (Green) and VPS4 (Red) signal during recruitment event onto HIV Gag-BFP VLP.
(TIF)

S2 Fig Model for different phases of ESCRT recruitment profile. The model shows how assembly and disassembly rates were measured by fitting the curves into Boltzmann Equation. This model also shows the different phases for ESCRT polymerization and de-polymerization as well as which part corresponds to calculating the retention time of ESCRTs.
(TIF)

S3 Fig Release of HIV Gag VLPs or full HIV Virions with NL4.3 backbone were not affected on tagged CHMP4B co-expression. HEK 293T cells were transfected with 1200 ng of CMV-eGFP-flex-CHMP4b and 800 ng of HIV Gag or 1800 ng HIV NL4.3 respectively. Cells and supernatant were analyzed by western blots 24hours later, and probed with GFP and p24 (left panels). No difference is seen in release of HIV Gag VLPs while a slight decrease is detected in release of HIV NL4.3 virions from cells expressing eGFP-flex-CHMP4b. Infectivity assay for harvested NL4.3 virions using luciferase assay in TZM-b1 cells 48 hrs post infection (right panel). Bar graphs show a slight reduction of infectivity comparable to the reduction in virion release observed in western blot analysis.
(TIF)

S4 Fig The raw western blots used in S3 Fig. (TIF)

S5 Fig The raw western blots used in S3 Fig. (TIF)

S6 Fig The raw western blots used in S3 Fig. (TIF)

S1 File (DOCX)
