# Supplementary figures and images for: High-speed imaging of ESCRT recruitment and dynamics during HIV virus like particle budding
Source: PLoS One. 2020 Sep 4;15(9):e0237268. doi: 10.1371/journal.pone.0237268 (PMC7473513; doi:10.1371/journal.pone.0237268)

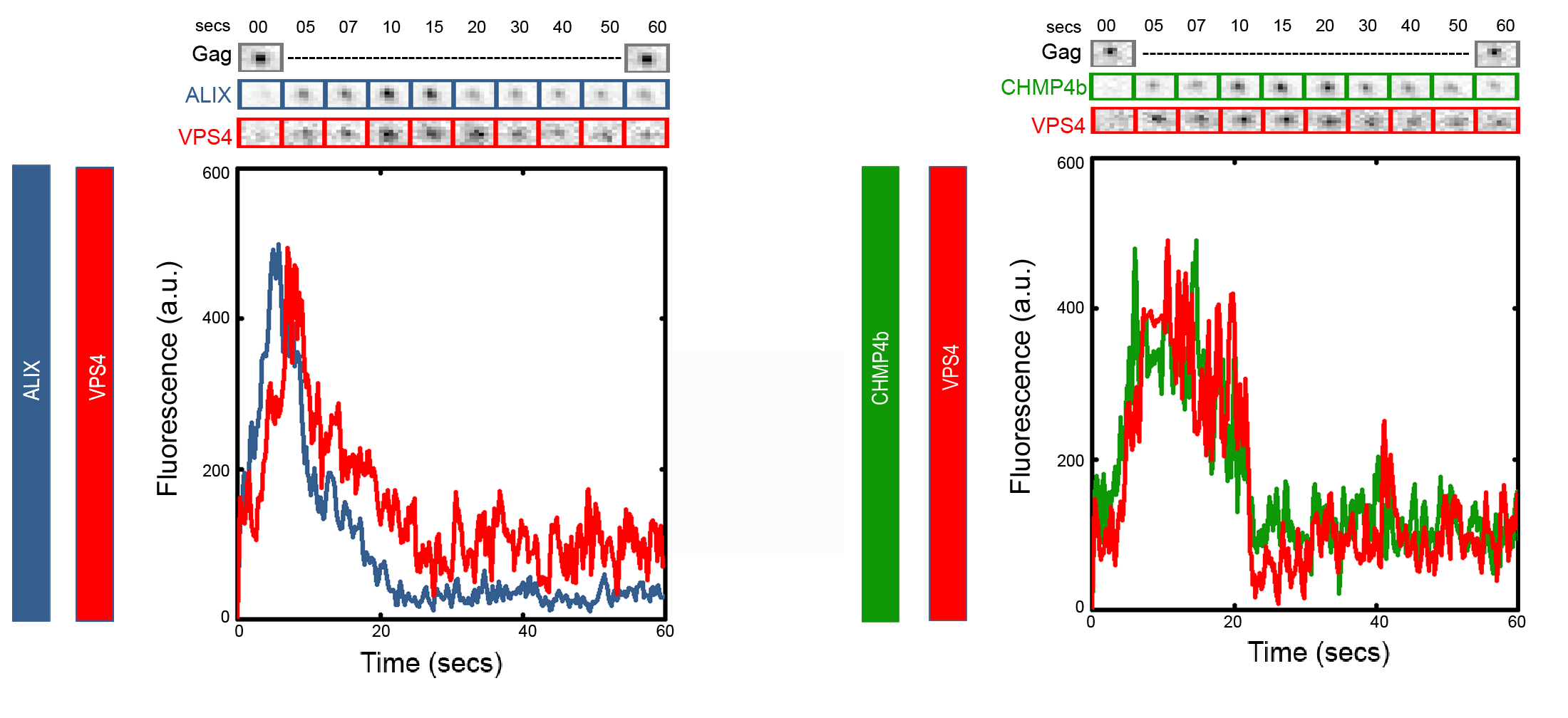

Supplement: S1 Fig — Left panel shows ALIX (Blue) and VPS4 (Red) signal during recruitment event onto HIV Gag-BFP VLP. Right panel shows CHMP4B (Green) and VPS4 (Red) signal during recruitment event onto HIV Gag-BFP VLP. (TIF) [file pone.0237268.s001.tif]

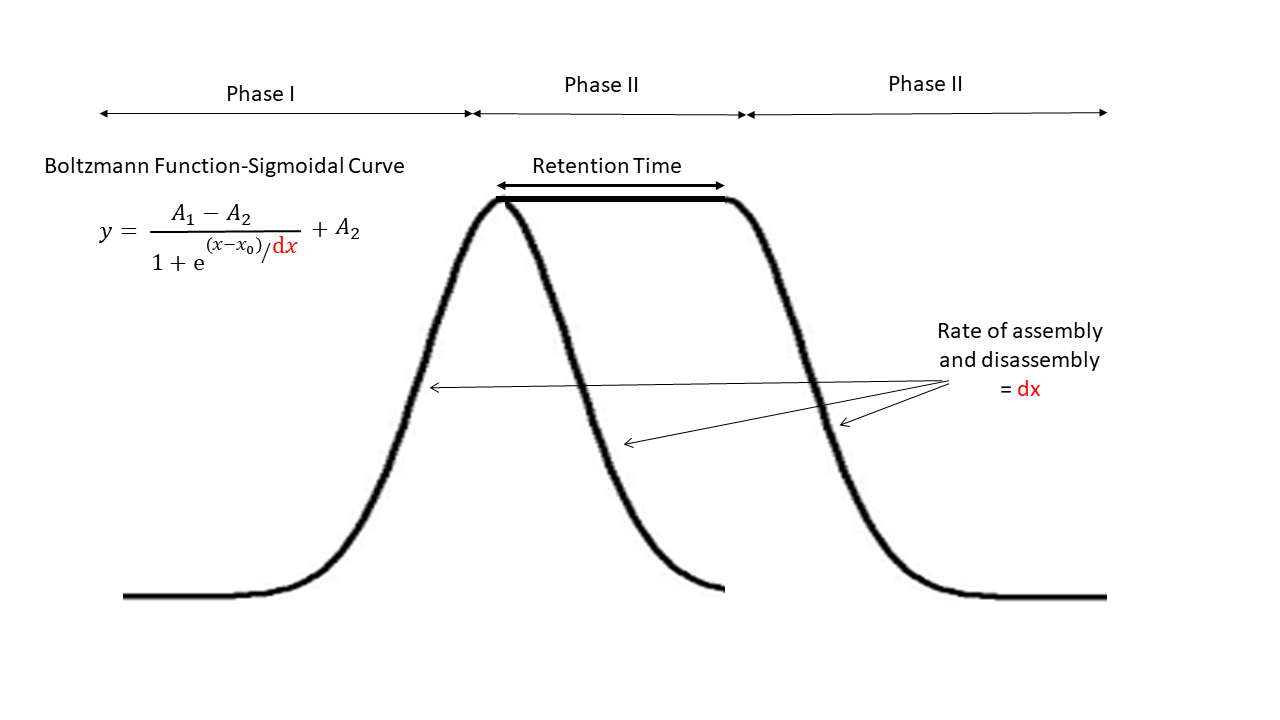

Supplement: S2 Fig — The model shows how assembly and disassembly rates were measured by fitting the curves into Boltzmann Equation. This model also shows the different phases for ESCRT polymerization and de-polymerization as well as which part corresponds to calculating the retention time of ESCRTs. (TIF) [file pone.0237268.s002.tif]

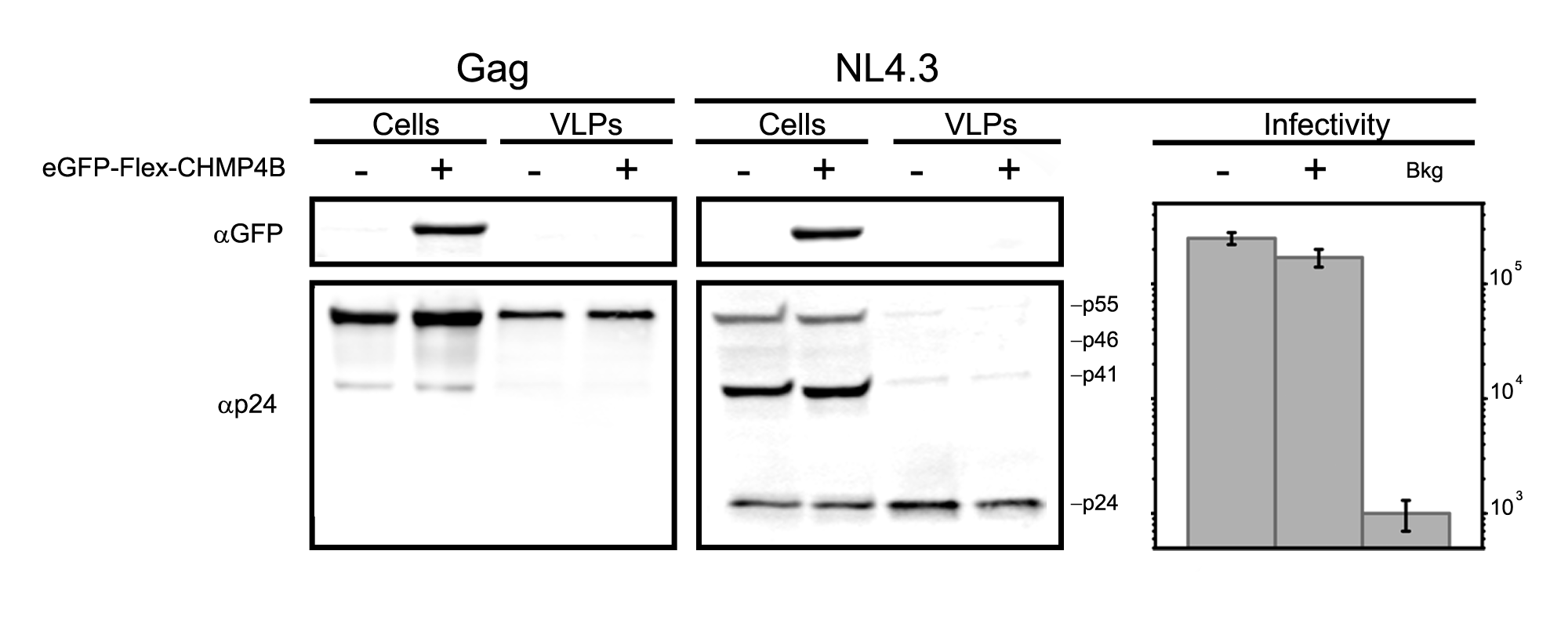

Supplement: S3 Fig — HEK 293T cells were transfected with 1200 ng of CMV-eGFP-flex-CHMP4b and 800 ng of HIV Gag or 1800 ng HIV NL4.3 respectively. Cells and supernatant were analyzed by western blots 24hours later, and probed with GFP and p24 (left panels). No difference is seen in release of HIV Gag VLPs while a slight decrease is detected in release of HIV NL4.3 virions from cells expressing eGFP-flex-CHMP4b. Infectivity assay for harvested NL4.3 virions using luciferase assay in TZM-b1 cells 48 hrs post infection (right panel). Bar graphs show a slight reduction of infectivity comparable to the reduction in virion release observed in western blot analysis. (TIF) [file pone.0237268.s003.tif]

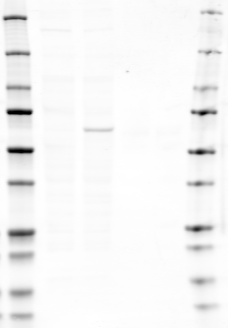

Supplement: S4 Fig — (TIF) [file pone.0237268.s004.tif]

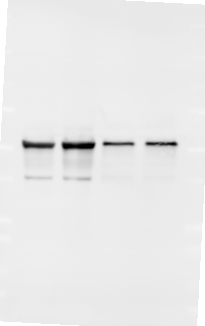

Supplement: S5 Fig — (TIF) [file pone.0237268.s005.tif]

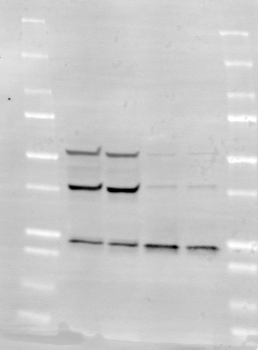

Supplement: S6 Fig — (TIF) [file pone.0237268.s006.tif]
